# The Structural Correlates of Statistical Information Processing during Speech Perception

**DOI:** 10.1371/journal.pone.0149375

**Published:** 2016-02-26

**Authors:** Isabelle Deschamps, Uri Hasson, Pascale Tremblay

**Affiliations:** 1 Département de Réadaptation, Université Laval, Québec City, QC, Canada; 2 Centre de Recherche de l’Institut Universitaire en santé mentale de Québec, Québec City, QC, Canada; 3 Center for Mind & Brain Sciences (CIMeC), University of Trento, Mattarello (TN), Italy; University of Barcelona, SPAIN

## Abstract

The processing of continuous and complex auditory signals such as speech relies on the ability to use statistical cues (e.g. transitional probabilities). In this study, participants heard short auditory sequences composed either of Italian syllables or bird songs and completed a regularity-rating task. Behaviorally, participants were better at differentiating between levels of regularity in the syllable sequences than in the bird song sequences. Inter-individual differences in sensitivity to regularity for speech stimuli were correlated with variations in surface-based cortical thickness (CT). These correlations were found in several cortical areas including regions previously associated with statistical structure processing (e.g. bilateral superior temporal sulcus, left precentral sulcus and inferior frontal gyrus), as well other regions (e.g. left insula, bilateral superior frontal gyrus/sulcus and supramarginal gyrus). In all regions, this correlation was positive suggesting that thicker cortex is related to higher sensitivity to variations in the statistical structure of auditory sequences. Overall, these results suggest that inter-individual differences in CT within a distributed network of cortical regions involved in statistical structure processing, attention and memory is predictive of the ability to detect structural structure in auditory speech sequences.

## Introduction

Auditory perception is a difficult task that involves the ability to process regularities (i.e. statistical structure) to make predictions about upcoming sounds. This ability relies, at least in part, on detecting the probabilities in which events co-occur [1, 2]. Transitional probabilities (TP) provide information regarding the regularity of auditory signals; this information can be used to predict upcoming sounds. The ability to detect TP is therefore a very important mechanism in the processing of auditory signals, both for language learning and language processing. In fact, the paradigmatic case is that of language acquisition during which children learn to use TP and other cues to segment speech into words, words into syllables, and syllables into phonemes. Indeed, from an early age, children are sensitive to TP in speech [3–6] and non-speech auditory signal [4]. Adults are also sensitive to TP in speech [7, 8] and non-speech auditory signals [4, 9–11], which suggests a general auditory competence, not specific to speech [4, 9–14]. Different underlying mechanisms have been proposed to account for these results. While some have proposed that TP and other statistics are computed/extracted online [4, 15] others have proposed that “chunking” mechanisms, dependent upon attention, memory and associative learning [16], underlie the apparent sensitivity to statistical structure. However, both approaches suggest that there is sensitivity to statistical regularities [17]. That is, the statistical structure of incoming auditory inputs must be detected.

Prior work using functional neuroimaging has identified the inferior frontal cortex (IFC), the superior temporal plane (STP), as well as subcortical regions (basal ganglia; BG) as sensitive to auditory statistical structure such as TP [18–27]. For example, regions within the frontal cortex, including the middle frontal gyrus (MFG) and the ventral premotor cortex (PMv), are sensitive to changes in perceived order in sequences of pure tones [18, 28], whereas the inferior frontal gyrus (IFG) is sensitive to TP in sequences of pure tones [22] and in syllable sequences in an artificial language [26]. Regions within the supratemporal plane are sensitive to TP in auditory tone sequences [22, 25], in bird songs [23] and in syllable sequences [21, 23, 29], as well as the sample entropy of pitch sequences [20]. Subcortical regions such as the BG are recruited during sequential structure learning using different modalities (e.g. visual, auditory and tactile) in which probabilistic relationships are manipulated [27, 30, 31].

While the relation between the processing of statistical structure and brain function has been studied extensively using fMRI, the relationship (if any) between brain anatomy and the processing of statistical structure remains poorly understood. Investigating this relationship might provide valuable insights into the underlying mechanisms involved in the processing of statistical structure, as gray matter is composed of neurons (including dendritic trees and spines), which are the building blocks in the transmission of information and brain functions. In fact, a recent review by Kanai and Rees [32] clearly demonstrates that brain morphometry has been repeatedly linked to inter-individual differences in numerous capacities (ranging from basic to higher cognitive functions). Specifically for language, two studies have demonstrated, using diffusion tensor imaging (DTI), that microstructural brain differences (i.e. integrity of white matter fiber tracts) predicted competencies often related to statistical learning; specifically, word-learning skills such as segmentation and rule learning [33, 34]. In these studies, increased fractional anisotropy or radial diffusivity was associated with better performance (i.e. less errors). Such results document a relationship between neurostructural differences and behavioral differences in performance on statistical learning abilities in healthy adults.

Other studies focusing on the more general relationship between brain structure and language or speech functions have also predominantly reported positive correlation between brain structure and performance. For example, with regards to auditory processing, it has been shown that musicians have greater CT in superior temporal surfaces than non-musicians [35]. In addition, relative pitch performance is predicted by higher CT in the bilateral intraparietal sulcus [36]. Other morphometric measures, such as white matter volume (WMV) and gray matter volume (GMV), also correlate with specific abilities. Successful learning of artificial grammars correlates with white matter integrity in left IFG (BA 44/45) [37]. In addition, successful learning of foreign speech sounds is correlated with higher WMV volume in the left Heschl’s gyrus (HG) [38] and bilaterally in a region anterior to the parietal-occipital sulcus [39], while successful learning of pitch patterns in word contexts is correlated with GMV in the left HG [40]. The structural differences observed in HG are in agreement with functional (i.e. increased activity) and structural differences (i.e. increase gray matter volume) that have been observed in individuals with good auditory perception, such as musicians [35, 41, 42]. Taken together, these studies demonstrate that structural brain imaging is a powerful tool for investigating the neural organization of auditory and language performance.

Expanding on our prior work [23], here we examined whether cortical thickness (CT) correlates with sensitivity to statistical structure in auditory sequences. In our previous fMRI study, we reported that during passive listening, several brain regions, mainly within the superior temporal plane, were sensitive to the statistical structure of auditory sequences. In contrast, here we focused on the relationship between CT, a measure of the depth of the cortical mantle [43, 44] and an explicit measure of sensitivity to statistical structure. Since a few prior studies have documented converging results between structural and functional neuroimaging ([33, 45, 46], but see also [47, 48]), here we expected that inter-subject variability in the ability to differentiate different levels of statistical structure would positively correlate with CT in regions including IFG, MFG, PMv, STP, and BG. We also expected to find positive correlations in regions not typically identified in fMRI studies of statistical structure processing, as the ability to detect/perceive different levels of statistical structure may also draw on cognitive processes such as working memory and attention [2, 16, 49] not necessarily involved in more implicit tasks.

## Materials and Methods

### Participants

Twenty healthy right-handed [50] native speakers of Italian (9 females; 24±4.5 years (range: 18–40), education: 17.1±3.64 years), with normal self-reported hearing, and no history of language or neurological/neuropsychological disorders participated in this study. We report data from 18 participants as two participants were removed because the FreeSurfer analyses were lost in a hardware crash and could not be replicated with the same computers and software versions. The study was approved by the Human research ethics committee of the University of Trento in Italy. Written consent was obtained from all participants.

### Stimuli and Procedures

The stimuli were sequences of speech and non-speech sounds. The individual sounds that were concatenated into sequences were recorded with a sampling rate of 44 kHz, and edited to have an envelope of 225 ms, ±15 ms fade in and fade out, and equal root mean square (RMS) intensity. A complete description of the stimuli can be found in Tremblay et al., [23]. The speech sounds were 70 Italian frequent consonant-vowel (CV) syllables. These syllables were composed of combinations of five vowels (/a, e, i, o, u/) and twenty-four consonants. A native male Italian speaker pronounced these syllables in a sound-attenuated booth; they were recorded directly to disk. The non-speech stimuli were 70 unique bird sounds created from a high quality digital collection of bird sounds recorded at 44 KHz, and commercially available on iTunes (The Ultimate Sound Effects Collection: Birds; 2010 by HDsoundFX). Bird sounds were used because, like speech, they form a natural class of spectrally complex sounds.

For each category of stimuli (speech, non-speech), sequences of 8.8 seconds were created with 3 different levels of statistical structure (low, mid, high). For each category, a set of 63 unique sequences was generated. Each sequence contained four different sounds that were repeated 8 times within individual sequence, resulting in sequences of 32 sounds. Within individual sequences, sounds were separated by 50 ms of silence and presented at a rate of 3.6 Hz. To optimize discriminability, each speech sequence included 4 maximally different (acoustically) consonants and vowels, and each bird sound sequence contained 4 sounds from different bird species.

Formally, the statistical structure of the sequences was determined by transition probability (TP) matrices, which were manipulated experimentally. The sequences ranged from random (i.e. lack of statistical structure) to highly structured in three levels (low, mid, high). Each level was associated with a different degree of Markov Entropy (ME). ME is a measure of unpredictability: the more predictable a sequence is, the lower its ME value. In the low structure condition, each item was equally likely to appear at any point independently of the previously presented item (mean±SD ME = 1.84±.03; range: 1.8–1.9). It was thus impossible for participants to form correct expectations. In the mid structure (mean±SD ME = 1.51±.018; range 1.48–1.53) and high structure sequences (mean±SD ME = .79±.012; range: 0.78–0.81), the TP matrices were more constrained, which allowed participants to form expectations about upcoming sounds (audio excerpts can be found in S1 Text). The overall proportion of self-repetitions was set at 25% within all three types of sequences. This was done to control for repetition suppression effects [51–53]. In addition, in all conditions the marginal frequencies of the four stimuli were identically set to 25%; thus the only differentiating factor were transition probability constraints (i.e., Shannon’s entropy was identical in all conditions, refer to Table 1). This experimental manipulation resulted in two within-subject factors: statistical structure (3 levels) and auditory category (2 levels).

**Table 1:** Transition probability matrix for the 3 levels of regularity.

**TP matrix used to construct the highly structured sequences(Markov Entropy = 0.81)**				
*From/To*	*Sound A*	*Sound B*	*Sound C*	*Sound D*
*Sound A*	25%	75%	0%	0%
*Sound B*	0%	25%	75%	0%
*Sound C*	0%	0%	25%	75%
*Sound D*	75%	0%	0%	25%
**TP matrix used to construct the highly semi-structure sequences (Markov Entropy = 1.49)**				
*From/To*	*Sound A*	*Sound B*	*Sound C*	*Sound D*
*Sound A*	25%	37.50%	37.50%	0%
*Sound B*	0%	25%	37.50%	37.50%
*Sound C*	37.50%	0%	25%	37.50%
*Sound D*	37.50%	37.50%	0%	25%
**TP matrix used to construct the highly semi-random sequences (Markov Entropy = 1.90)**				
*From/To*	*Sound A*	*Sound B*	*Sound C*	*Sound D*
*Sound A*	25%	25%	25%	25%
*Sound B*	25%	25%	25%	25%
*Sound C*	25%	25%	25%	25%
*Sound D*	25%	25%	25%	25%

### Behavioral task and responses

**Fig 1:**
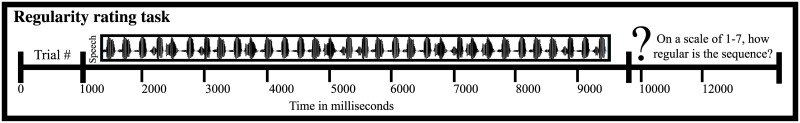
Experimental design. The trial number was displayed for 1000 ms and was followed by the an auditory sequence which lasted for 8800 ms. A question mark then appeared triggering participants to determine sequence regularity on a scale of 1 to 7.

Participants were first introduced to the syllables and bird sounds, to avoid any surprise effect during the main experiment and then they heard the 126 unique sequences during a passive listening task performed during a fMRI session (reported in [23]). Following the MRI session, participants were presented again with the same sequences while seated comfortably in front of a computer monitor, wearing a high quality headset through which the sound sequences were presented in random order using Presentation Software (Neurobehavioral Systems). On each trial, participants were asked to rate the degree of statistical structure (Regularity Sensitivity; RS) perceived in the sequences (scale of 1 [not structured] to 7 [highly structured]), and in 40% of all trials, they were also asked how many distinct sounds they perceived in the sequence, similarly rated using a 1–7 scale (See Fig 1). Participants were told that sequences containing repeating, predictable patterns (the following example PA-TA-KA-PA-TA-KA-PA-TA-KA was given) should be rated as highly structured (close to seven), while random, unpredictable sequences should be rated low (close to one). Here, we focus on the analysis of statistical structure ratings (RS) because it offers insights into participants’ ability to detect changes in statistical structure in speech and non-speech sound sequences—i.e., to successfully differentiate between series solely on the basis of the transition constraints between sounds. Given that, to our knowledge, the RS scale has not been used in prior work, we assessed its internal consistency for each level of statistical structure (high, mid, low) for speech and non-speech sounds separately using a split-half reliability procedure [54]. That is, within each auditory category, for each level of statistical structure, we separated the odd trials from the even trials. We then calculated the split half coefficient for each level of statistical structure. The rating scale has good internal consistency for each level of statistical structure for speech and non-speech sounds (speech: high α = .95, mid α = .95, low α = .93; non-speech: high α = .73, mid α = .93, low = .89).

For each auditory category (i.e. speech and non-speech) we used a repeated measures ordinal logistic regression (rmOLR) at the group level to determine whether participants’ RS ratings could be predicted from the three levels of statistical structure (i.e. high, mid, low). First, for each participant, we calculated their average rating for each of the three levels of statistical structure (high, mid, low). Then, the average rating obtained for each statistical structure level was rounded to the nearest integer. Before running the rmOLR, we tested the assumption of collinearity using a linear regression with the ratings of statistical structure as the dependent variable and the actual level of statistical structure (high, mid, and low) as the independent variable. We found no violation of this assumption (Speech High: Tolerance = 0.789, *VIF* = 1.267; Speech Mid: Tolerance = 0.789, *VIF* = 1.267; Non-speech High: Tolerance = 0.789, *VIF* = 1.267; Non-speech Mid: Tolerance = 0.789, *VIF* = 1.267). Next we verified whether the assumption of proportional odds was met using a full likelihood ratio test comparing the residual of the fitted location model to a model with varying location parameters, (speech: χ^2^(4) = 9.033, *p* = 0.340; non-speech: χ^2^(4) = 1.710, *p* = 0.789).

A rmOLR was then performed to evaluate sensitivity to statistical structure separately for the speech and bird-song, with participants’ RS ratings as the dependent variable and statistical structure levels (3) as the within-subject independent variable. For the speech sequences, the results from the rmOLR indicated a strong relation between participants’ ratings and statistical structure, Wald *χ*^2^(2) = 19.688, *p* = 0.00005. This was shown statistically in that the odds ratio for being in a higher category of the dependent variable (i.e. higher rating on the 7 point scale) for the high versus low level of statistical structure was 34.34 (95% CI [7.2, 163.82]), which was statistically significant, Wald *χ*^2^(1) = 19.68, *p* = 0.000009. Similarly, the odds ratio of being in a higher category of the dependent variable for the mid versus low level of statistical structure and for the high versus mid level of statistical structure were also statistically significant (mid-low odds ratio = 1.839, 95% CI [1.02, 3.29], Wald *χ*^2^(1) = 4.17, *p* = 0.041; high-mid odds ratio = 18.68, 95% CI [4.59 76.07], Wald *χ*^2^(1) = 16.7, *p* = 0.00004). To summarize, for the speech sequences, participants’ ratings tracked the level of regularity, and differentiated the high from mid-regularity levels as well as the mid from low-regularity levels.

For the non-speech sequences, the results from the rmOLR indicated that the level of actual statistical structure influenced the ratings, Wald *χ*^2^(2) = 13.93, *p* = 0.001. However, we found that participants appeared to discriminate the high regularity from the other two conditions, but did not discriminate the mid-regularity and low-regularity conditions. Statistically, this was seen in that the odds ratio of being in a higher category of the dependent variable was only statistically significant for the high versus low level of statistical structure (odds ratio = 3.73, 95% CI [1.78, 7.79], Wald *χ*^2^(1) = 12.22, *p* = 0.0005) and high versus mid level of statistical structure (odds ratio = 3.07, 95% CI [1.43, 6.56], Wald *χ*^2^(1) = 8.31, *p* = 0.004). For the mid versus low level of statistical structure the odds ratio of being in a higher category of the dependent variable was not significant (odds ratio = 1.216, 95% CI [0.58 to 2.57], Wald *χ*^2^(1) = 0.261, *p* = 0.61). Thus, unlike for the speech sequences, for the non-speech sequences participant did not appear to detect the difference in statistical structure between the mid versus low level. Because of this weaker sensitivity to statistical structure in bird-song sequences, we excluded the bird songs from the morphometry analyses. The remaining analyses therefore focus on the relationship between CT and the sensitivity to statistical structure in speech sequences.

**Fig 2:**
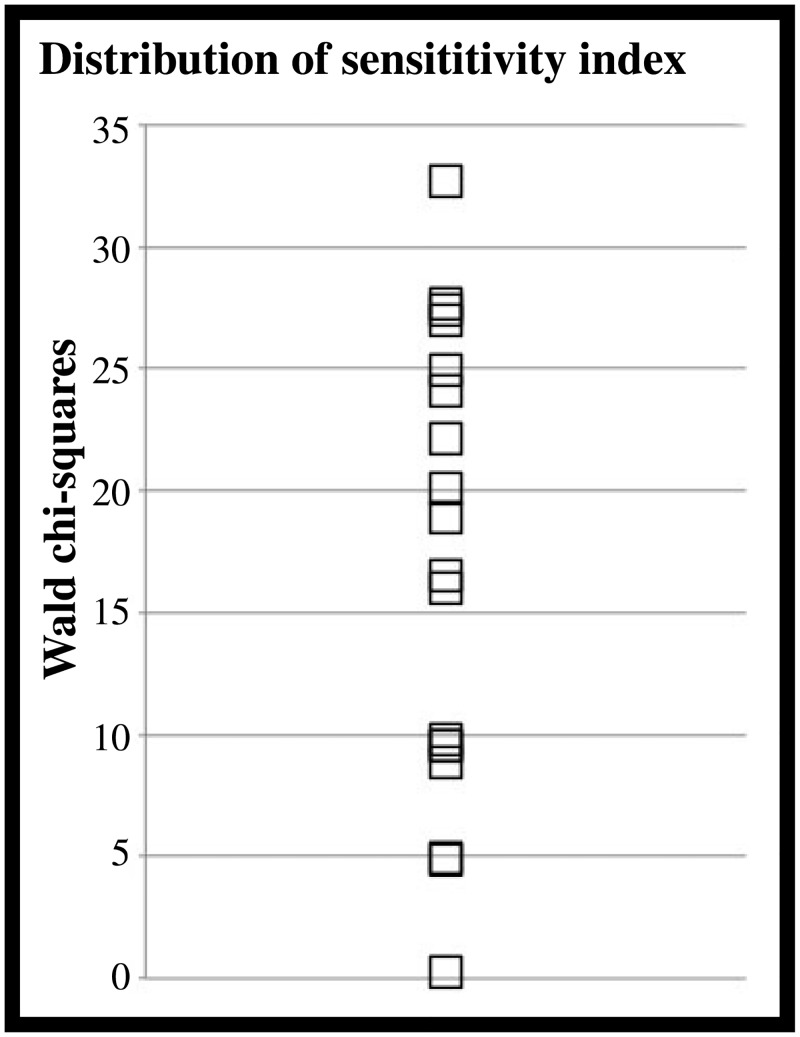
Distribution of sensitivity coefficients for speech sounds. A. Scatterplot illustrating the distribution of the Wald chi-square statistics for speech.

After establishing that, at the group level, participants could perceive differences in statistical structure among the 3 levels for the speech sequences, we derived one index per participant quantifying the degree to which changes in statistical structure were associated with changes in ratings. For each participant we calculated, the degree to which ratings predicted the actual level of statistical structure of a sequence. An ordinal logistic regression (OLR) with ratings as the dependent variable and statistical structure levels (3) as the independent variable was computed for each participant. This procedure returned a Wald Chi-Square statistics per participant (the higher the Chi-Square, the more participant’s ratings predicted the level of statistical structure of the speech sequences). After verifying that the distribution of the Wald Chi-Square statistics across all subjects was normal, these statistics were used as predictors in the brain-behavior analysis that forms the core of this study (See Fig 2).

### Image acquisition

Two T1-weighted 3D MPRAGE structural images were acquired [1x1x1 mm3, GRAPPA iPAT = 2, 5:36min each]. One was optimized for optimal contrast [MPRAGE_CNR] between gray and white matter tissue [TE/TR/TI/flip angle = 4.18ms/2700ms/1020ms/7°] and the other was optimized for signal to noise ratio [MPRAGE_SNR] in gray and white matter tissue [TE/TR/TI/flip angle = 3.37 ms / 2500 ms /1200 ms / 12°]. The two were co-registered and averaged to obtain a single high quality structural image per participant.

### MRI Image analysis

#### Pre-processing of the T1-images

CT measures and subcortical volumetric brain measures were obtained using Freesurfer v5.3.0 [55–57]. First, a surface representation of each participant’s anatomy was created by inflating each hemisphere of the averaged anatomical volume to a surface representation. The resulting surface representation was aligned to a template of average curvature. These surface representations were obtained by submitting each participant’s structural image to a series of steps that included: (1) motion correction (across the two volumes) and affine transformation to Talairach space, (2) intensity normalization, (3) removal of non-brain voxels using skull stripping algorithms, (4) segmentation of gray and white matter as well as cerebrospinal fluid and (5) surface tessellation. At each step, intermediate results were inspected and manual interventions were performed when required to correct topological assignment errors. These included mainly manual annotations to establish white-matter/gray-matter boundaries (control-point intervention) and manual removal of residual dura mater or skull. The surface representations were then parcellated using an automated parcellation scheme [57, 58]. This scheme relies on a probabilistic algorithm that incorporates the anatomical convention of Duvernoy [59]. The anatomical accuracy of this method is high and approaches the accuracy of manual parcellations [57, 60, 61].

#### Investigation of the relationship between brain morphometry and behavior

Our main objective was to examine the relationship between CT and sensitivity to statistical structure. This was accomplished in two ways: (1) by conducting a whole-brain vertex-wise analysis correlating CT with sensitivity to statistical structure, (2) by examining the relationship between subcortical volumes in relation to sensitivity to statistical structure (these were not included in the cortical-surface analysis in #1) and (3) by investigating whether mean CT measures within functionally-defined regions of interest (ROIs) correlated with sensitivity to statistical structure. We report the analyses in that order.

#### Whole-brain analyses

Whole-brain CT measures were obtained from FreeSurfer routines calculating the closest distance from the gray/white boundary to the gray/CSF boundary at each vertex on the tessellated surface [43]. The CT maps were created using spatial intensity gradients across tissue classes and are therefore not simply reliant on absolute signal intensity. We exported the individual maps to SUMA (AFNI’s 3D cortical surface mapping module; [62]) where we conducted group-level analyses on the surface. Prior to the group-level analyses, we smoothed the individual CT data using a 10-mm full-width-at-half-maximum Heat kernel.

For the whole-brain CT measures, we tested, using a vertex-wise linear regression model (AFNI covariate analysis option in the 3dttest++ program), whether CT correlated with the statistical sensitivity index that we derived for each participant. Age was included in the analysis as a covariate to control for the well-established relation between age and CT [63, 64]. We also conducted an additional vertex-wise linear regression model in which sex was included as an additional covariate. This analysis is detailed in S2 Text. The resulting group maps were corrected for multiple comparisons using the Monte Carlo simulation procedure implemented in FreeSurfer. Only areas in which CT significantly correlated with the statistical sensitivity index were included in the final corrected maps (individual vertex threshold of p < 0.05, corrected for multiple comparisons to achieve a whole-brain family-wise error (FWE) rate of p < 0.05 (clusters ≥ 437 vertices)).

#### Subcortical volumes

To examine subcortical structures, we used the automated procedure for volumetric segmentation implemented in FreeSurfer. This procedure relies on the segmentation of subcortical structure, which is based on voxel intensity, spatial comparisons with a probabilistic training atlas as well as comparisons to neighboring voxel labels [61]. For each bilateral subcortical structure (thalamus, caudate, putamen, pallidum, hippocampus, amygdala, nucleus acumbens and cerebellum), a volume measurement was obtained from the native anatomical data. FreeSurfer morphometric procedures have been demonstrated to show good test-retest reliability across scanner manufacturers and across field strengths [65]. For each subcortical volume, we then examined whether sensitivity to statistical structure predicted volume using a multiple regression model in which the sensitivity index, age and intracranial volume were included as predictors. We also conducted additional multiple regressions in which sex was also included as a predictor. This analysis is detailed in S2 Text. The resulting p-values were corrected for multiple comparisons using a False Discovery Rate (FDR) procedure (q* = 0.05, i = 16, 2 hemispheres x 8 ROIs).

#### Functional ROIs

**Fig 3:**
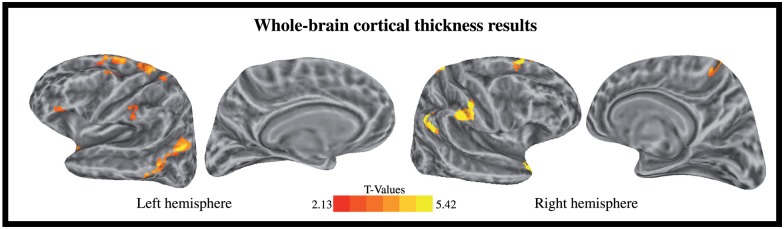
Regions showing a significant relationship between CT and sensitivity to statistical structure. Results are shown on the group-average inflated white matter inflated surface. All analyses are controlled for multiple comparisons.

To further explore the relationship between CT and sensitivity to statistical structure, we created a set of seven masks based on the functional data reported [23]. Masks were based on the regions that showed a significant effect of statistical structure (Fig 3A; [23]). For each participant, the average thickness of each of the 7 clusters was extracted. For each ROI, we then examined whether sensitivity to statistical structure predicted CT using a multiple regression model in which the sensitivity index, and age were included as predictors. We also conducted additional multiple regressions in which the sensitivity index, age and sex were included as predictors. This analysis is detailed in S2 Text. The resulting p-values were corrected for multiple comparisons using a False Discovery Rate (FDR) procedure (q* = 0.05, i = 7).

## Results

### Whole-brain results

**Fig 4:**
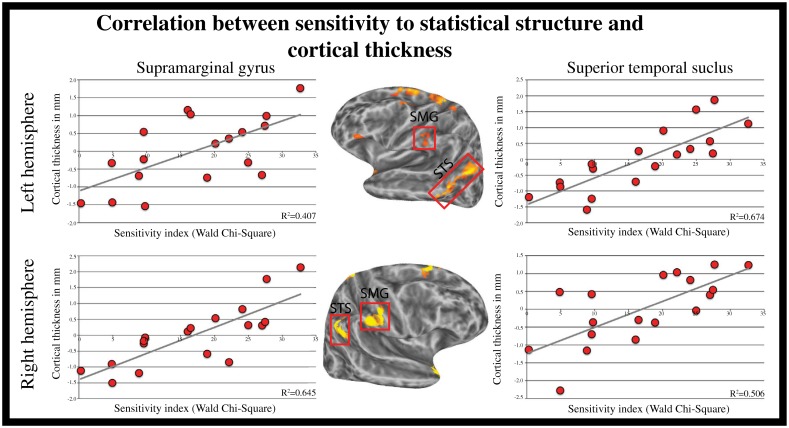
Scatter plot illustrating the relationship between CT and sensitivity to regularity with age regressed out (the values plotted are the resulting z-transformed residuals). SMG = supramarginal gyrus; STS = superior temporal sulcus.

The whole-brain analysis targeted cortical regions in which CT correlated with participant’s sensitivity to statistical structure. We identified several regions showing such a correlation, and importantly, all correlations were positive: thicker cortex was associated with a higher sensitivity to statistical regularities in auditory syllable sequences. We found such correlations in the bilateral SMG, the bilateral angular gyrus/superior temporal sulcus (AG/STS), the bilateral superior frontal gyrus and sulcus (SFG/S), the superior portion of the left precentral sulcus (PrCS), the left postcentral gyrus and central sulcus (PoCG/CS), the ventral portion of the anterior insula (vAI), the left precentral sulcus and MFG (PrCS/MFG), the IFG pars triangularis and anterior insula (IFGpt/AI), the right temporal pole and anterior portion of the superior temporal gyrus (STGa), and the right paracentral gyrus and sulcus (Refer to Figs 3 and 4 and Table 2, for a complete list).

**Table 2:** Clusters where the correlation between CT and sensitivity to statistical structure was statistically significant.

Anatomical location	Hemi	x	y	z	T-value	P-value	Number of nodes	Area(mm2)
Angular gyrus, superior temporal sulcus and middle temporal gyrus	Left	-47	-69	19	5.427	0.00007	1501	394.52
Superior portion of the precentral sulcus	Left	-20	-20	60	4.277	0.0007	1001	319.17
Superior frontal gyrus and sulcus	Left	-18	8	59	5.162	0.0001	607	201.29
Postcentral gyrus and central sulcus	Left	-22	-33	65	4.175	0.0008	713	170.01
Anterior ventral insula	Left	-36	-1	-19	4.185	0.0008	775	147.9
Precentral sulcus and middle frontal gyrus	Left	-29	-4	44	4.499	0.0004	462	134.44
Inferior frontal gyrus pars triangularis and anterior insula	Left	-50	25	3	4.987	0.0002	599	121.6
Supramarginal gyrus and postcentral sulcus	Left	-59	-25	26	4.009	0.0011	450	82.7
Supramarginal gyrus and postcentral gyrus	Right	57	-24	32	8.51	0.0000004	1362	285.56
Temporal pole and lateral superior temporal gyrus	Right	42	24	-28	4.161	0.0001	689	229.81
Paracentral gyrus and sulcus and postcentral sulcus	Right	10	-40	74	3.821	0.0016	914	197.45
Superior frontal gyrus and sulcus	Right	12	25	54	3.481	0.0034	528	170.33
Angular gyrus and superior temporal sulcus	Right	49	-53	34	5.231	0.0001	1064	148.19

All coordinates are in Talairach space and represent the maximum surface node value for each of the cluster (minimum cluster size: 437 contiguous surface nodes, each significant at p < .05).

### Analyses of subcortical volume and functional ROIs

No relationship was found between GMV and sensitivity to statistical structure in any of the subcortical structures even at an uncorrected threshold. In addition, no relationship was found between CT and sensitivity to statistical structure in any of the functional ROIs even at an uncorrected threshold.

## Discussion

This is the first study to investigate the potential relationship between cortical thickness in healthy adults and the ability to detect statistical structure in non-deterministic sequences of speech sounds. Our main finding is that the thickness of several cortical areas correlates positively with sensitivity to statistical structure in auditory syllable sequences.

Given that the cellular and molecular mechanisms that determine CT are complex, and that structural MRI cannot identify the source of CT variations (e.g. increased neuronal or synaptic density, increased density in glial cells), the link between brain anatomy and performance can be interpreted in several ways. Neuronal changes that could affect CT include neurogenesis, synaptogenesis, increased dendritic formation, neuronal morphology, and neuronal arrangement, as well as the natural processes of synaptic pruning [66, 67]. In the present study, the relation between CT and sensitivity to statistical structure was positive indicating that thicker cortex was associated with better performance (i.e. better ability to perceived differences between statistical structures). More specifically, the whole-brain analyses revealed that increased sensitivity to statistical structure was associated with thicker cortex in regions that have been previously associated with the processing of statistical structure using fMRI (i.e. bilateral AG/pSTS, left PrCG, left MFG), as well as regions typically associated with cognitive functions such as memory and attentional processes (bilateral SMG, bilateral SFG\S, left vAI). These findings are discussed in the following paragraphs.

### Behavior/CT correlations in areas implicated in statistical processing

Among the regions in which CT co-varied with participant’s sensitivity to statistical structure for speech, the left ventral IFGpt, the left MTG, the left PrCS, and the bilateral STS are regions that have previously been linked to the processing of statistical information in auditory and language stimuli [22, 28, 29, 68]. In particular, prior work suggests that the involvement of the left ventral IFG might be related to the processing of auditory sequential information. For example, Nastase [22] reported that the left ventral IFG, a region similar to the one reported in the current study, differentiated between levels of disorders in auditory sequences of pure tones. Clusters of activation within this region have also been reported during musical sequence processing [69, 70] and rule learning in an artificial language [33, 71]. Taken together, these findings are consistent with the notion that the left ventral IFG might provide a neural basis for processing statistical information based on statistical cues (i.e. TP) present in auditory signals. One possibility is that in the current study, the left ventral IFG relied on TP for the construction of predictions regarding upcoming information in speech sequences.

In addition, we also found a positive correlation between CT and sensitivity to statistical structure in the left MFG, a region similar to the one reported in Tobia et al., [18] during the active monitoring of order change in tone sequences. Interestingly, this region was not recruited during the passive detection of order changes in tone sequences [18] or during the passive detection of statistical structure in speech and non-speech sounds [23]. This suggests that attentional demands modulate the network of brain regions involved in the detection of statistical structure. The correlation between CT and sensitivity to statistical structure within the left MFG supports the involvement of this region in the explicit evaluation of statistical structure, but not during passive processing. We also observed a positive correlation in the bilateral STS, a region that has been associated with the ongoing computation of transitional probabilities [21, 29]. McNealy et al., [21, 29] have linked the superior temporal plane (including STS) to the processing of statistics. In sum, in the current work, which focused on the structural correlates associated with the explicit assessment of statistical structure, our results identified a subset of regions that are typically associated with the processing of statistical information using fMRI.

### Behavior/CT correlations in areas implicated in attentional and memory processes

Interestingly, our results reveal correlations between CT and brain areas traditionally implicated in working memory (e.g., bilateral ventral SMG, SFS) [72, 73] and attentional processes (vAI) [74–76]. For instance, prior work has documented the involvement of the SMG during the manipulation of sequences of phonemes/syllables in working memory [77, 78] and during the learning of an artificial language [21, 29]. In experiments investigating the automatic processing of auditory syllable sequences with different levels of statistical structure, and in experiments investigating non-verbal auditory sequences, SMG activation is not typically documented [18, 22, 23], suggesting that the anterior SMG is involved in maintaining phonological information in memory [79]. This hypothesis is supported by the present finding of a relationship between CT and the processing of sequences of syllables, as the processing of syllable sequences requires the updating of phonological information in working memory as the sequence unfolds over time. Similarly, we also found a significant correlation in the bilateral SFS. This might suggest a close relationship between working memory and the ability to detect changes in statistical structure, given that the bilateral SFS/G is engaged in working memory [80, 81].

In addition, within the left vAI, thicker CT was associated with greater sensitivity to statistical structure. While left vAI is not usually associated with the processing of statistical information, it is a region that is active across a variety of tasks encompassing a broad range of processing domains [82, 83], including speech [84], interoceptive awareness [85], and emotions [86], and it has been associated with the ventral attention network in studies examining responses to oddball stimuli [87]. It has been suggested that the anterior insula plays a role in domain-general attention control [76, 88]. Thus, one possibility is that our finding for this region reflects inter-individual attentional differences whereby individuals with thicker CT would be better at focusing their attention and, as a result, would be better at detecting differences in statistical structure among the three levels of statistical structure. This view is congruent with the notion that the ability to detect changes in statistical structure relies, at least partly, on other cognitive functions such as attention and memory [2, 16, 49]. Future studies are needed to clarify the relationship between attention, memory and statistical information processing.

### Absence of Behavior/CT correlations in areas sensitive to statistics during passive listening

We note that we did not find correlations between CT and sensitivity to statistical structure within the functional ROIs based on our previous work, which identified regions in which brain activation tracked statistical structure during passive listening. Some of these regions (the right medial transverse temporal gyrus and the bilateral medial transverse temporal sulci) only tracked statistics only tracked statistical structure in *non*-speech sounds, while our dependent measure here was derived from sensitivity to speech sounds (see section Behavioral tasks and responses for the rationale). However, in our previous work, other regions of the primary auditory cortex, the bilateral lateral transverse temporal gyrus and sulcus and the medial transverse temporal gyrus, tracked statistical structure regardless of auditory category. Yet, in the present study, we found no correlation between cortical thickness and sensitivity to statistical structure within these regions. This lack of correspondence between functional and morphometric data may reflect the fact that the crux of computations of TP takes place on higher cortical areas of the processing hierarchy. For instance, one possibility is that A1 receives afferent inputs after regularity has been coded by higher-level regions (e.g. SMG, STS). The predictions generated in these higher-level regions would then be evaluated against inputs in A1. Hence, while A1 would not necessarily code for statistical structure, the predictions generated by these higher-level regions would still have an impact on the activity in A1 (top-down effect). This is congruent with a recent study by Tobia et al., [28] in which the authors found afferent connectivity from the superior temporal sulcus to the left A1 during the perception of sequences of tones.

Another possible explanation is that regions involved in the processing of statistical information as identified using fMRI do not necessarily correlate with structural differences within these regions. This is consistent with several studies in the literature investigating brain structure-function relationships. For instance, Hoeft et al., [48], used fMRI to compare brain activation associated with visual rhyme judgments in dyslexic adolescents, age-matched and reading-matched controls. In the dyslexic adolescents, hypoactivations were found within the left inferior parietal lobule (IPL) and the bilateral fusiform/lingual gyri as compared to controls. Interestingly, the hypoactivation in left IPL was the only one that was accompanied by significant differences in grey matter volume between dyslexic adolescents and age-matched controls (i.e. smaller grey matter volume in dyslexic adolescents). All other regions of hypoactivations (i.e. bilateral fusiform) had no relation to brain structure. Similarly, Nyberg et al., [89] reported that while many cortical regions exhibited less activation as a function of age during a semantic categorization task, this pattern observed could only be explained in terms of grey matter volume differences in one of these regions (i.e. right mid-frontal cortex). For the other regions, no relationship between brain structure and function was found. Relatedly, Kadosh et al., [90] found variations in brain activation as a function of age during a facial expression detection task in the left fusiform gyrus and the right inferior temporal gyrus. These variations in brain activation correlated with white matter volume (WMV) differences in the left fusiform gyrus and the right inferior temporal gyrus as well as grey matter volume (GMV) volume differences in the left fusiform gyrus. A positive correlation between brain activation and age was also found during a gaze detection task within the left supramarginal gyrus. This correlation was not related to differences in grey or white matter volume in that region. In addition, Tremblay et al., [47] demonstrated that most age differences in task-related BOLD signal magnitude during speech tasks are not related to neurostructural changes. Taken together, these results suggest that functional activation is not systematically related to brain structure, or to specific morphometric measures (e.g. CT, GMV, WMV).

Alternatively, it is also possible that the neural mechanisms associated with passive listening to statistical regularities (as assessed in our previous fMRI study) are distinct, at least in part, from the mechanisms underlying active assessment of statistical regularity (as assessed in the present study). Therefore, while in both studies the same auditory sequences were used, the nature of the tasks differed (active vs. passive). One possibility is that the brain regions identified here are ones that utilize attention and memory to process speech sounds; these regions might not show differential responses to different levels of regularity (as more attentive individuals may do so independent of regularity), but their structure could co-vary with one’s ability to discriminate levels of regularity. Another possibility is that the regions identified in the functional study are involved in performing low-level ‘chunking’ operations that are linked to the transition probability structure during online listening, but do not strongly contribute to the explicit evaluation of overall input disorder.

Overall, our results support the idea that the ability to detect differences in statistical structure in auditory sequences relies on several different perceptual, cognitive and executive mechanisms including attention and memory, in addition to mechanisms more directly related to statistical structure processing. This idea is supported by the observation that most of the regions in which CT correlated with sensitivity to statistical structure have been described by Chen et al. [91] as being part of three different modular CT correlations networks (among the six modules that were proposed) (see [91, 92], for a recent review). These studies demonstrate that CT organization follows a modular network structure matching specific functions such as auditory and language processing. In the current study, we observed significant correlations within regions involved in higher-level cognitive functions (executive/strategic; Module 1), sensorimotor/spatial functions (Module 2) and auditory processing/language functions (Module 5) [91]. While sensitivity to statistical structure appears to rely, to a large extent, on domain-general neural resources, the results of the current study cannot distinguish between models that seek to differentiate the numerous mechanisms underlying sensitivity to statistical structure. As such, our results are congruent both with models that posit that sensitivity to structure is the end result of computing transition probabilities [15] as well as with models that propose that sensitivity to statistical structure is a by-product of “chunking” mechanisms that rely on memory, attention and associative learning [16].

## Conclusion

We detected inter-individual differences in sensitivity to statistical structure in sub-lexical speech sequences using a behavioral procedure, and at the whole-brain level using vertex morphometry. In addition to showing, for the first time, that sensitivity to statistical structure can be investigated using surface-based brain morphometry, we also show that the neural system identified are dominantly cortical and include both specialized and domain-general areas. This suggests that domain-general regions not specific to auditory or statistical processing support the ability to detect statistical structure, a mechanism that may play an important part in our day-to-day life, allowing us to form expectations about future events and using this information to interact more efficiently with our complex environments. More studies are needed to replicate our findings using larger sample size, and to gain further insights into the complex relationship between brain structure, brain functions, and the processing of statistical information.

## Supporting Information

S1 Fig Cortical thickness results corrected for sex. A. Red: sensitivity to statistics controlled for age and sex. Yellow: sex effects controlled for age and sensitivity to statistics. B. Overlap between sex and statistical effects.
(EPS)

S1 Text (PDF)

S2 Text (DOCX)
