# Supplementary material for: The Structural Correlates of Statistical Information Processing during Speech Perception
Source: PLoS One. 2016 Feb 26;11(2):e0149375. doi: 10.1371/journal.pone.0149375 (PMC4771024; doi:10.1371/journal.pone.0149375)
Supplement: S1 Text — (PDF) [file pone.0149375.s002.pdf]

S1. Text

A. **BirdR1.wav.** Bird-song sequence random structure

B. **BirdM1.wav.** Bird-song sequence mid-structure

C. **BirdH1.wav.** Bird-song sequence high-structure

D. **SpeechR1.wav.** Syllable sequence random structure

E. **SpeechM1.wav.** Syllable sequence mid-structure

F. **SpeechH1.wav.** Syllable sequence high-structure
