# Supplementary material for: The Structural Correlates of Statistical Information Processing during Speech Perception
Source: PLoS One. 2016 Feb 26;11(2):e0149375. doi: 10.1371/journal.pone.0149375 (PMC4771024; doi:10.1371/journal.pone.0149375)
Supplement: S2 Text — (DOCX) [file pone.0149375.s003.docx]

**S2 Text**

In order to account for the relationship between CT and sex [1], we conducted additional analyses. These analyses are detailed below.

### Whole-brain analyses

The whole-brain CT measures obtained via the analysis pipeline reported in the main text constituted the predicted variable here. For the whole-brain CT measures, using a vertex-wise linear regression model (AFNI covariate analysis option in the 3dttest++ program), whether (1) CT correlated with the statistical sensitivity index that we derived for each participant, while controlling for age and sex, (2) CT correlated with sex while controlling for age and sensitivity to statistical structure. The resulting group maps were corrected for multiple comparisons using the Monte Carlo simulation procedure implemented in FreeSurfer (individual vertex threshold of p < 0.05, corrected for multiple comparisons to achieve a whole-brain family-wise error (FWE) rate of p < 0.05 (clusters ≥ 437 vertices)).

In addition, we also identified whether regions in which CT varied as a function of the statistical sensitivity index overlapped with regions in which CT varied as a function of sex via a conjunction mask of brain activity [2]. The conjunction mask was achieved by computing the intersection of the corrected t-maps for (1) the effect of sex on CT while controlling for age and sensitivity to statistical structure, and (2) the effect of the sensitivity to statistical structure while controlling for age.

### Subcortical volumes

The volume measures obtained via the analysis pipeline reported in the main text constituted the predicted variable here. For each subcortical volume, we then examined whether sensitivity to statistical structure predicted CT using a multiple regression model in which the sensitivity index, age, total intracranial volume and sex were included as predictors. The resulting p-values were corrected for multiple comparisons using a False Discovery Rate (FDR) procedure (q*=0.05, i=16, 2 hemispheres x 8 ROIs).

### Functional ROIs

To further explore the relationship between CT and sensitivity to statistical structure, we created a set of seven masks based on the functional data reported in Tremblay et al., [3]. Masks were based on the regions that showed a significant effect of statistical structure (Panel A of Figure 3; [3]). For each participant, the average thickness of each of the 7 clusters was extracted. For each ROI, we then examined whether sensitivity to statistical structure predicted CT using a multiple regression model in which the sensitivity index, age and sex were included as predictors. The resulting p-values were corrected for multiple comparisons using a False Discovery Rate (FDR) procedure (q*=0.05, i=7).

**Results**

**Whole-brain analyses**

The first whole-brain analysis targeted cortical regions in which CT correlated with participant’s sensitivity to statistical structure while controlling for age and sex. We identified several regions in which a positive relationship between CT and sensitivity to statistical structure was found. Correlations were found in the bilateral angular gyrus/superior temporal sulcus (AG/STS), the left precentral gyrus and sulcus (PrCS/G), the left inferior occipital gyrus and sulcus/middle occipital gyrus, the left ventral portion of the anterior insula (vAI), the right supramarginal gyrus (SMG), and the right temporal pole/superior temporal gyrus (TP/STG; Refer to S1 Fig panel A and S1 Table, for a complete list).

**S1 Table: Clusters where the correlation between CT and sensitivity to statistical structure was statistically significant while controlling for age and sex.**

| **Anatomical location** | **Hemi** | **x** | **y** | **z** | **T-value** | **P-value** | **Number**  **of nodes** | **Area(mm2)** |
| --- | --- | --- | --- | --- | --- | --- | --- | --- |
|  |  |  |  |  |  |  |  |  |
| Angular gyrus, superior temporal sulcus and middle temporal gyrus | Left | -48 | -72 | 24 | 5.766 | 0.000049 | 1273 | 320.51 |
| Precentral sulcus and gyrus | Left | -20 | -20 | 60 | 3.706 | 0.002349 | 617 | 199.58 |
| Inferior occipital gyrus and sulcus and middle occipital gyrus | Left | -41 | -83 | -15 | 4.234 | 0.000834 | 506 | 181.69 |
| Postcentral gyrus | Left | -22 | -33 | 65 | 3.791 | 0.001986 | 494 | 122.31 |
| Anterior ventral insula | Left | -36 | -1 | -19 | 3.72 | 0.002285 | 659 | 119.35 |
| Inferior frontal gyrus pars triangularis | Left | -50 | 25 | 3 | 4.635 | 0.000386 | 529 | 116.02 |
| Supramarginal gyrus, postcentral gyrus and central sulcus | Right | 57 | -24 | 31 | 8.339 | 0.000001 | 1060 | 210.97 |
| Angular gyrus and superior temporal sulcus | Right | 49 | -53 | 34 | 4.656 | 0.000371 | 1226 | 177.25 |
| Temporal pole and lateral superior temporal gyrus | Right | 39 | 14 | -31 | 3.721 | 0.002281 | 537 | 177.22 |

**All coordinates are in Talairach space and represent the maximum surface node value for each of the cluster (minimum cluster size: 437 contiguous surface nodes, each significant at p < .05).**

**[S1 Fig about here]**

**S1 Fig. Whole brain correlates of cortical thickness for sensitivity to statistical structure.** Panel A. Regions in dark red represent areas in which a positive relationship between sensitivity and statistical structure after controlling for age and sex was found. Regions in yellow represent areas in which a relationship between sex and CT was found after controlling for age and sensitivity to statistical structure. Panel B. The region in red represents the only area showing an overlap between regions in which sex effects were observed after controlling for sensitivity to statistical structure and age and regions in which sensitivity to statistical structure was observed after controlling for age. Results are shown on the group-average inflated white matter inflated surface. All analyses are controlled for multiple comparisons using a cluster extent of 437 vertices, and a single node threshold of p < 0.05, to achieve a family-wise error rate of p < 0.05.

The second whole-brain analysis targeted cortical regions in which CT correlated with sex while controlling for age and sensitivity to statistical structure. The correlation between sex and CT was more circumscribed that the one found between sensitivity to statistical structure and CT. A relationship between CT and sex was found in the left paracentral gyrus/precentral gyrus, the left middle frontal gyrus (MFG), the right PrCG, the right vAI, the right superior parietal gyrus (SPG) and the PoCG/S (Refer to S1 Fig panel A and S2 Table, for a complete list).

**S2 Table: Clusters where the correlation between CT and sex was statistically significant while controlling for age and sensitivity to statistical structure.**

| **Anatomical location** | **Hemi** | **x** | **y** | **z** | **T-value** | **P-value** | **Number of nodes** | **Area(mm2)** |
| --- | --- | --- | --- | --- | --- | --- | --- | --- |
|  |  |  |  |  |  |  |  |  |
| Paracentral gyrus and sulcus and precentral gyrus | Left | -10 | -30 | 71 | 3.374 | 0.004541835 | 523 | 157.33 |
| Middle frontal gyrus | Left | -39 | 11 | 49 | 4.544 | 0.000458923 | 537 | 102.83 |
| Precentral gyrus | Right | 12 | -24 | 72 | 5.273 | 0.00011781 | 2083 | 618.61 |
| Anterior insula and orbital gyrus | Right | 34 | 14 | -3 | 4.211 | 0.000871705 | 469 | 141.28 |
| Superior parietal gyrus and precuneus | Right | 20 | -59 | 60 | 3.846 | 0.001781542 | 672 | 139.8 |
| Postcentral gyrus and sulcus | Right | 21 | -34 | 66 | 2.837 | 0.013183585 | 450 | 85.53 |

**All coordinates are in Talairach space and represent the maximum surface node value for each of the cluster (minimum cluster size: 437 contiguous surface nodes, each significant at p < .05).**

The results of the conjunction analysis demonstrate that regions in which sex effects were observed after controlling for sensitivity to statistical structure and age do not overlap with regions in which sensitivity effects after controlling for age were observed in the right hemisphere. In the left hemisphere, overlap between changes in CT as a function of sensitivity to statistical structure and changes in CT as a function of sex was found in a very small cluster located in the posterior middle frontal gyrus (refer to S1 Fig panel B).

1. Sowell ER, Peterson BS, Kan E, Woods RP, Yoshii J, Bansal R, et al. Sex differences in cortical thickness mapped in 176 healthy individuals between 7 and 87 years of age. Cerebral cortex. 2007;17(7):1550-60. doi: 10.1093/cercor/bhl066. PubMed PMID: 16945978; PubMed Central PMCID: PMCPMC2329809.

2. Nichols T, Brett M, Andersson J, Wager T, Poline J-B. Valid conjunction inference with the minimum statistic. NeuroImage. 2005;25(3):653-60.

3. Tremblay P, Baroni M, Hasson U. Processing of speech and non-speech sounds in the supratemporal plane: Auditory input preference does not predict sensitivity to statistical structure. NeuroImage. 2012;66C:318-32. Epub 2012/11/03. doi: 10.1016/j.neuroimage.2012.10.055. PubMed PMID: 23116815.
